# Reproducibility of PD patient-specific midbrain organoid data for *in vitro* disease modeling

**DOI:** 10.1016/j.isci.2025.113541

**Published:** 2025-09-10

**Authors:** Elisa Zuccoli, Haya Al Sawaf, Mona Tuzza, Sarah L. Nickels, Alise Zagare, Jens C. Schwamborn

**Affiliations:** 1 Developmental and Cellular Biology, Luxembourg Centre for Systems Biomedicine (LCSB), University of Luxembourg, 6, Avenue du Swing, 4367 Belvaux, Luxembourg

**Keywords:** molecular biology, Neuroscience, cell biology, omics

## Abstract

Midbrain organoids are advanced *in vitro* cellular models for disease modeling. They have been used successfully over the past decade for Parkinson’s disease (PD) research and drug development. The three-dimensional structure and multicellular composition allow disease research under more physiological conditions than is possible with conventional 2D cellular models. However, there are concerns in the field regarding the organoid batch-to-batch variability and thus the reproducibility of the results. In this manuscript, we generate multiple independent midbrain organoid batches derived from healthy individuals or glucocerebrosidase (GBA)-N370S mutation-carrying PD patients to evaluate the reproducibility of the GBA-N370S mutation-associated PD transcriptomic and metabolic signature as well as selected protein abundance. Our analysis shows that GBA-PD-associated phenotypes are reproducible across organoid generation batches and time points. This proves that midbrain organoids are not only suitable for PD *in vitro* modeling but also represent robust and highly reproducible cellular models.

## Highlights

• Midbrain organoids provide a robust *in vitro* model for Parkinson’s disease
• GBA-PD phenotypes are reproducible across midbrain organoid batches
• NESC passage has a greater impact on variability than organoid batches
• Disease signatures are consistent at transcriptomic, protein, and metabolic levels

## Graphical abstract

**Figure:**
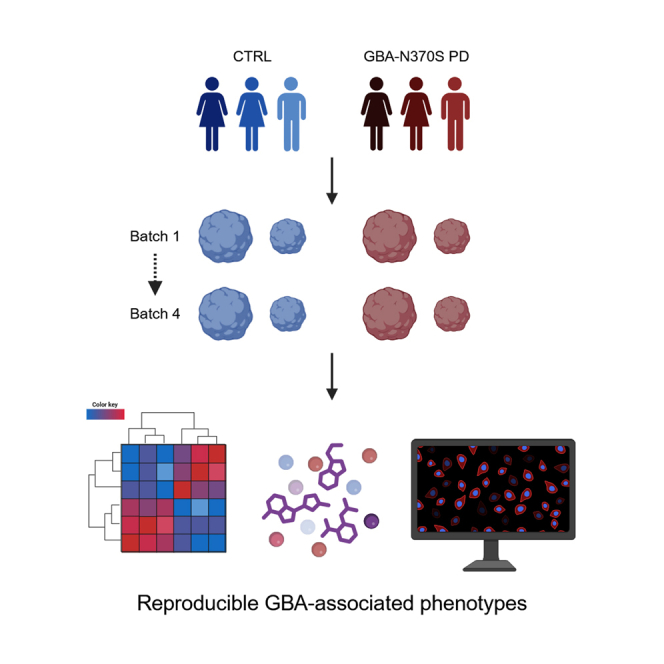

## Introduction

Parkinson’s disease (PD) is a common neurodegenerative disorder characterized by the progressive loss of midbrain dopaminergic neurons, leading to motor symptoms such as bradykinesia, rigidity, and tremors, as well as cognitive impairments.^1^ Conventional two-dimensional (2D) cell cultures and animal models have limitations in replicating the complexity of the human midbrain, hindering their utility in understanding PD pathogenesis, and developing effective therapies.^2^ Specifically, 2D cultures lack intricate cell-to-cell interactions, which are present in three-dimensional structures, and are crucial for accurately modeling neural environments. Additionally, animal models, such as mice, do not naturally develop neurodegenerative diseases like Parkinson’s, and their brain anatomy differs significantly from humans, which limits their effectiveness in studying complex human brain disease processes.^3^ Recent breakthroughs in stem cell technologies have resulted in the development of three-dimensional (3D) midbrain organoids, which more closely mimic the architecture and cellular diversity of the human midbrain. Importantly, these organoids are derived from patient-specific induced pluripotent stem cells (iPSCs) and are generated via the differentiation of iPSCs into neuroepithelial stem cells (NESCs), which subsequently self-organize into midbrain-like structures.^4^^,^^5^ This approach offers a physiologically relevant platform to study disease mechanisms and to test therapeutic interventions on patient-specific genetic backgrounds. Glucocerebrosidase (GBA)-associated PD, linked to mutations in the GBA (GBA1) gene, represents one of the most significant genetic risk factors for PD.^6^ GBA mutations impair lysosomal function and autophagy, contributing to α-synuclein protein accumulation and dopaminergic neuron loss.^6^ Additionally, cellular senescence has emerged as a contributing factor in PD, with evidence suggesting that senescent cells exacerbate neurodegeneration through pro-inflammatory pathways.^7^^,^^8^^,^^9^ Midbrain organoids derived from GBA-PD patients have shown hallmarks of disease, including dopaminergic neuron loss, oxidative stress, impaired lipid metabolism, and a senescence-associated phenotype.^10^ These findings underline the value of midbrain organoids as a robust platform to explore the interplay between genetic risk factors, cellular senescence, and neurodegeneration. iPSC-derived organoid models hold the potential to inform therapeutic strategies targeting the underlying mechanisms of GBA-associated PD and related neurodegenerative disorders.

Although midbrain organoid models hold significant promise, they also present certain limitations that must be carefully considered when designing experiments. Complex, multistep culturing protocols can increase sources of variability, thus increasing the number of replicates needed both within a batch and between independent batches. Therefore, it is important to be aware of variation sources in order to account for it. In this manuscript, we explore these sources of variation and inform on the relevance of cell passage in reducing batch-to-batch variability. Overall, our results show that key disease phenotypes are reproducible despite potential sources of variability.

## Results

### Disease and cell passage are key sources of variation in midbrain organoid culture

**Figure 1:**
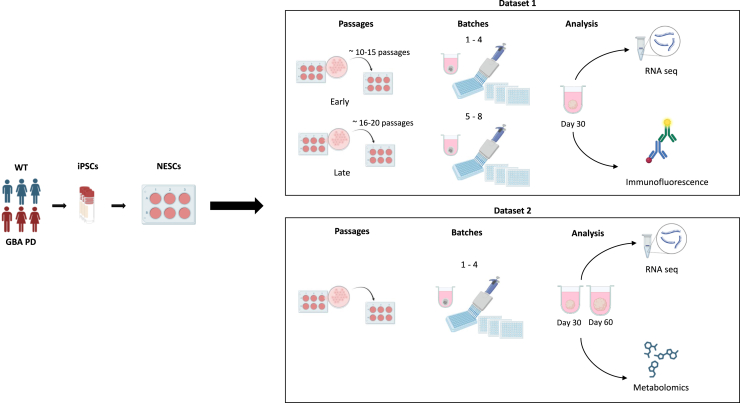
Overview of factors affecting midbrain organoid culture and dataset generation Schematic overview of the factors affecting midbrain organoid culture and dataset generation. iPSCs from healthy control (WT) and GBA-PD patients were differentiated into NESCs. For dataset 1, midbrain organoids from early (∼10–15) or late (∼16–20) passages were generated in four independent batches. On day 30 of midbrain organoid culture, RNA-seq and immunofluorescence experiments were performed. For dataset 2, four independent batches of midbrain organoids were generated. On day 30 or day 60 of midbrain organoid culture, RNA-seq and metabolomic analysis were performed.

The midbrain organoid culture system is influenced by several factors, including cell line selection, initial passage number, donor sex, and potential batch effects from different experimental runs, all of which require careful consideration (Figure 1; Table 1). To investigate each factor’s contribution to the data variance, we generated midbrain organoids from three healthy donors and three patients with PD carrying the GBA-N370S variant (GBA-PD) (Table S1). iPSCs derived from GBA-PD patients and healthy donors were differentiated into NESCs and patterned toward a midbrain identity to generate midbrain organoids.^4^ In this study, two independent datasets were produced, where dataset 1 comprised RNA sequencing (RNA-seq) and immunofluorescence analyses performed on 30-day-old midbrain organoids and dataset 2 included metabolomic analysis and RNA-seq analysis performed on both day 30 and day 60 midbrain organoids cultures. While dataset 1 was used to define experimental and biological sources of variation, dataset 2 served to validate the reproducibility of key phenotypes across independent experiments (Figure 1).

**Table 1:** Nomenclature

Statistical term	Culture term	Definition
Biological replicate	individual	healthy or GBA-N370S mutation-carrying donor
	patient	donor with Parkinson’s disease (PD) diagnosis
	cell line	an iPSC cell line is a population of pluripotent stem cells derived from a single donor through one reprogramming event. Multiple independent iPSC lines (clones, see below) from the same donor can be generated as biological replicates to account for variability
	passage	cell line passages refer to the number of times a cell culture is split (propagated) once it becomes confluent
	clone	a clone is a population of genetically identical cells derived from a single parent cell, typically originating from a specific donor; multiple clones can be derived from a single donor, but a single clone is often selected for experiments to ensure genetic and phenotypic consistency
	batch	each batch represents independent generations of midbrain organoids from different neuroepithelial stem cell (NESC) passages, accounting for variability in organoid maturation; within a batch, organoids serve as biological replicates, cultured and processed together under identical conditions to minimize batch effects and ensure reproducibility
	sample	a sample is a unique experimental unit used for analysis. Its origin depends on the experiment and assay, coming from either an independent organoid culture (batch), a pool of organoids within the same batch, or multiple batches (batch pooling); a sample is represented as a single data point in the analysis of an experiment
Technical replicate	section	sections obtained from a single organoid using a vibratome are considered technical replicates, i.e., repeated measurements of the same biological sample; they help to assess the reproducibility of the equipment and protocol, minimizing technical variability and error; unlike biological replicates, which capture variation between independent organoid cultures, technical replicates ensure differences are due to biological effects rather than inconsistencies in sample processing or analysis

**Figure 2:**
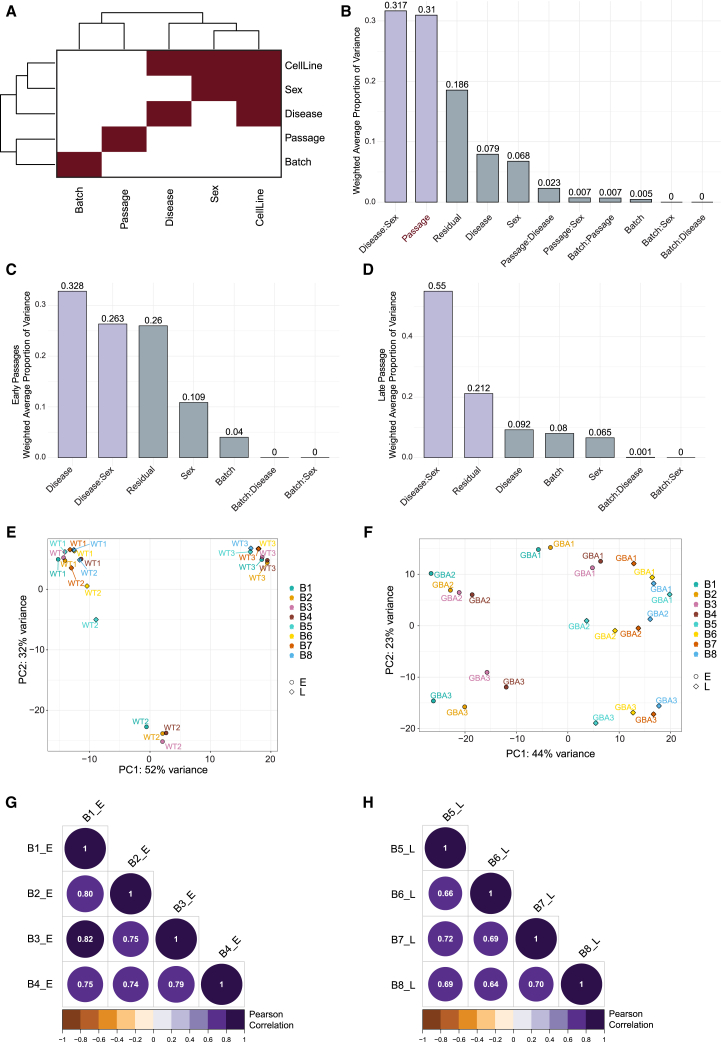
PVCA identifies the disease and the passage to be the major sources of variation in midbrain organoid culture Midbrain organoids were generated at early or late passage in four independent batches and transcriptomic analysis was performed. (A) Correlation analysis of variance features including batch, passage, disease, sex, and cell line. (B) Principal variance component analysis (PVCA) of transcriptomic data including all passages. (C) PVCA of early passage transcriptomic data. (D) PVCA of late passage transcriptomic data. (E) Principal-component analysis (PCA) on healthy control (WT) samples from early and late passage and all eight batches. (F) PCA on PD-GBA samples from early and late passage and all eight batches. (G) Pearson correlation of log2 fold change (FC) of significant genes (*p* < 0.05) between batches derived from early-passage NESCs. (H) Pearson correlation of log2 FC of significant genes (*p* < 0.05) between batches derived from late-passage NESCs.

Correlation analysis of transcriptomic signatures of eight independent organoid generation batches from early and late NESC passages revealed that the features such as disease, cell line, and donor sex are interdependent factors, representing the donor (patient or healthy individual). However, the passage of NESCs and the organoid generation batch are independent sources of variance (Figure 2A). Applying principal variance component analysis (PVCA), a statistical method for quantifying and prioritizing sources of variance, revealed that the interaction between disease and sex (31.7%) contributed most to the variance in the transcriptomic data. The passage (31%) appeared as the second highest contributor, followed by the residual variance (18.6%), which represents the variance that cannot be attributed to specific explanatory variables or known factors in our dataset. The variance attributed to batch (5%) or its interaction with passage (0.7%), sex (0%), or disease (0%) showed an insignificant role in the data variance (Figure 2B).

Considering the presumed role of the passage number in transcriptomic data variation, we divided the dataset in batches generated from early (pE10–15) or late NESC passages (pL16–20) to explore the effects of passage on data reproducibility in detail. The PVCA showed that the disease accounted for the largest proportion of the variance (32.8%) in organoid batches generated from early-passage NESCs, followed by the interaction between disease and sex (26.3%) and the unexplained residuals (26%). The percentage of the batch was elevated compared to the complete dataset (4%) (Figure 2C). Similarly, the disease-sex interaction with 55% was the highest contributor to the variance in the organoid batches produced from the late passages (B5–8), followed by the residual variance (21.2%). We observed that the proportion of variance of the batch doubled in organoids generated from late-passage NESCs compared to the early-passage NESCs (8%) (Figures 2D and S1A), indicating that the increased batch-to-batch variability is due to the use of NESCs at later passage numbers during organoid generation. Next, we investigated the effects of passages and batches on each cell line’s individual transcriptomic profile. Using principal-component analysis (PCA), we observed that organoid samples from healthy donors (wild type [WT]) tended to cluster separately, demonstrating biological variance, which is independent of passage. Only in the WT2 cell line, we observed an effect of passage, with WT2 progressively resembling the WT1 cell line with each passage. This indicates that the WT2 cell line may exhibit greater variability in the phenotype depending on the NESC passage (Figure 2E). In contrast, GBA-PD midbrain organoid samples clustered rather depending on the initial NESC passages, indicating that patient samples are more sensitive to experimental design and culture conditions (Figures 2F and S1B).

To explore the passage effect on a disease signature, we correlated the expression of significantly differentially expressed genes (DEGs) between healthy controls and GBA-PD midbrain organoids generated in batches from early or late NESC passages. Early-passage batches (B1–4) showed a significant correlation of DEG expression (0.74–0.82) (Figure 2G), while late-passage batches (B5–8) showed on average a moderate correlation (0.64–0.72) (Figure 2H). Similarly, looking at the expression of all shared genes across the late- or early-passage batches, we saw a higher (0.51–0.64) correlation for the early-passage batches than for the late-passage batches (0.42–0.50) (Figures S1C and S1D).

Overall, exploratory analysis of potential sources of variation in the transcriptomic data of midbrain organoids suggests that disease status and number of passages are major sources of gene expression variance, while batch effects are minimal. Moreover, early passages show more consistent transcriptomic profiles, while late passages lead to increased variability, emphasizing the need to consider NESC passage in study design.

### Reproducible transcriptomic disease signature across independently generated datasets

The passage number showed a more considerable impact on data reproducibility than organoid batches. We further wanted to assess more in detail how the choice of NESC passages used for organoid generation might influence the reproducibility of disease phenotypes.

**Figure 3:**
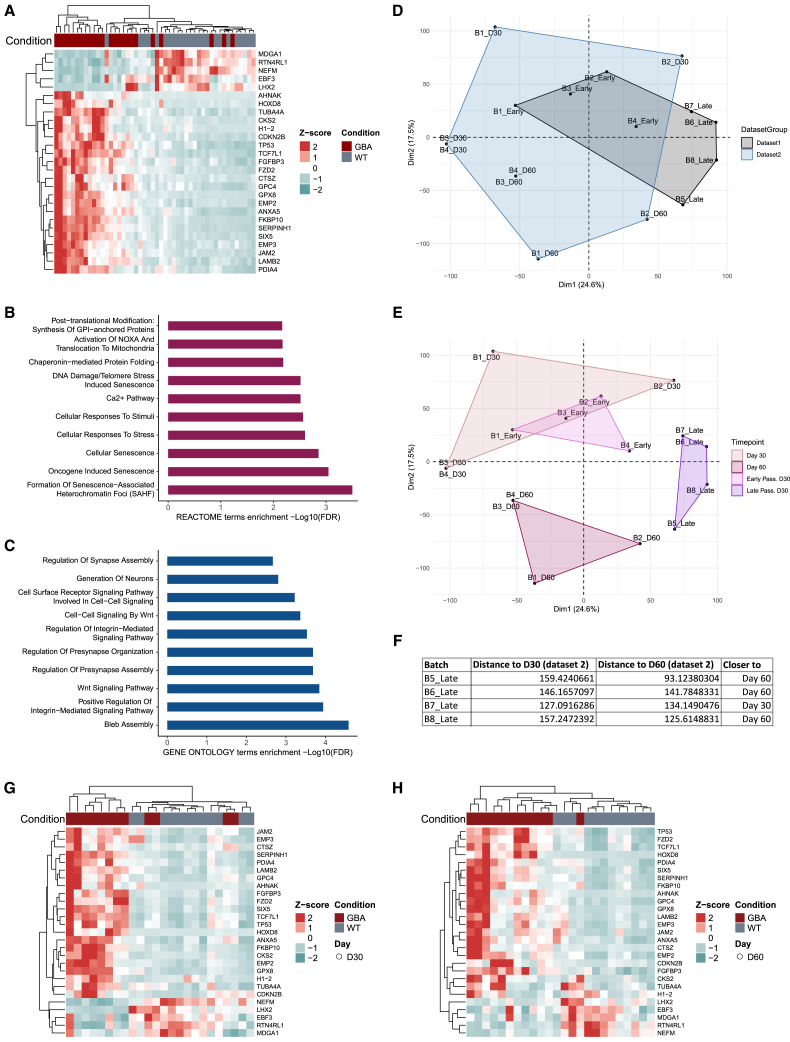
Disease signature is reproducible across independent datasets at the transcriptomic level For dataset 1, midbrain organoids were generated at early or late passage in four independent batches, and transcriptomic analysis was performed. For dataset 2, midbrain organoids were generated in four independent batches at two time points, and transcriptomic analysis was performed. (A) Unsupervised hierarchical clustering of GBA-PD and healthy control (WT) samples based on normalized gene counts of 27 predefined genes of all passages. (B) REACTOME pathway enrichment analysis of the DEGs between GBA-PD and healthy control (WT) samples. (C) Gene Ontology pathway enrichment analysis of the DEGs between GBA-PD and healthy control (WT) samples. (D) Integration of log2 fold change (FC) from dataset 1 and dataset 2 shown in principal-component analysis (PCA) plot. Samples are clustered by dataset. (E) Integration of log2 FC from dataset 1 and dataset 2 shown in PCA plot. Samples are clustered by time point and passage. (F) Euclidean distance table showing the batch, the calculated distance to day 30 or day 60, and the proximity to one of the two time points. (G) Unsupervised hierarchical clustering of GBA-PD and healthy control (WT) samples based on normalized gene counts of 27 predefined genes at day 30. (H) Unsupervised hierarchical clustering of GBA-PD and healthy control (WT) samples based on normalized gene counts of 27 predefined genes at day 30.

We grouped the early-passage batches (B1–4) and the late-passage batches (B5–8) and identified the significant DEGs within these data subsets. We found 27 overlapping DEGs in the early-passage set, whereas the late-passage batches did not show shared DEGs (Figures S2A and S2B). This could be due to the lower gene expression correlation between organoid batches from late-passage NESCs, representing higher data variability. Since the DEG expression signature at early passages appeared more consistent across organoid batches, we presumed 27 DEGs shared among the early-passage batches as a potential key GBA-PD disease signature and examined its reproducibility in both early- and late-passage batches. Here, we observed that the clustering of WT versus GBA-PD samples for the 27 DEGs was notably more distinct in the early-passage heatmap compared to the late-passage heatmap (Figures S2C and S2D), where we still observed adequate separation. Moreover, by combining all eight batches, the expression of selected 27 DEGs also separated WT and GBA-PD samples, indicating the relevance of these genes in disease pathogenesis (Figure 3A). Enrichment analysis of the 27 significant DEGs between GBA-PD and WT samples revealed an association with Reactome pathways related to cellular senescence and apoptosis, consistent with findings of recent studies (Figure 3B).^10^ Additionally, a Gene Ontology enrichment analysis supported these results and identified “bleb assembly,” which is linked to apoptosis, as the most enriched pathway, further confirming the disease signature in GBA-PD (Figure 3C). Next, we classified the 27 genes into distinct functional categories. These included the senescence/apoptosis pathway, biological processes related to synapse and nervous system development, metabolic and oxidative stress processes, cell adhesion and extracellular matrix dynamics, and transcription and gene regulation. Importantly, the fold change (FC) of GBA-PD vs. WT demonstrated a highly similar dysregulation trend for all genes across all eight batches (Figure S2E).

The initial dataset included organoid transcriptomic data from a single time point—day 30 of organoid culture. We produced new independent organoid batches and performed RNA-seq and metabolomic analyses for organoids at two time points, day 30 and day 60 (dataset 2). These analyses were done to additionally assess how reproducible the phenotypes are at different time points of organoid culture and across independent experiments. The transcriptomic analysis allowed us to compare the two datasets, where the PCA showed, as expected, a tighter clustering for dataset 1 (day 30 organoids) compared to a broader pattern in dataset 2 (day 30 and day 60 organoids) (Figure 3D). Early passage samples (B1–4_Early) from dataset 1 clustered with day 30 samples from dataset 2 (B1–4_D30), showing a similar expression profile (Figure 3E). In contrast, late passage samples (B5–8_Late) from dataset 1 formed a separate cluster, highlighting the significant impact of passage number on gene expression (Figure 3E). To assess the similarity of specific data points to predefined clusters, we calculated the Euclidean distance of individual points from the centroids of designated groups. The analysis revealed that most late passage samples (B1–4_Late) aligned more closely with day 60 samples (B5–8_D60), except for “B7_Late,” which clustered near day 30 (B1–4_D30) (Figure 3F). These findings indicate that late-passage midbrain organoids display phenotypes more similar to those observed in longer term midbrain organoid cultures, which may reflect age-related changes in the organoid system. Next, we wanted to determine whether the samples in the newly generated dataset shared the same disease signature defined by the 27 significant DEGs common to the early-passage NESC organoid batches. We observed that the WT and GBA-PD samples showed an even clearer clustering at day 60 compared to day 30. This finding aligns with expectations as day 60 midbrain organoids are more aged, leading to the accumulation of cellular, epigenetic, and transcriptional changes that enhance the distinction between sample groups (Figures 3G and 3H).

Altogether, our analysis shows that while NESC passage can influence phenotype detection, the disease signature identified in early passages is reproducible in later passages and across independent sequencing experiments from two distinct organoid culturing time points. This establishes the midbrain organoid model as a reliable tool for disease phenotyping and modeling.

### Validation of dopaminergic neuron and senescence phenotypes at the protein level confirms the transcriptomic disease signature in midbrain organoids

**Figure 4:**
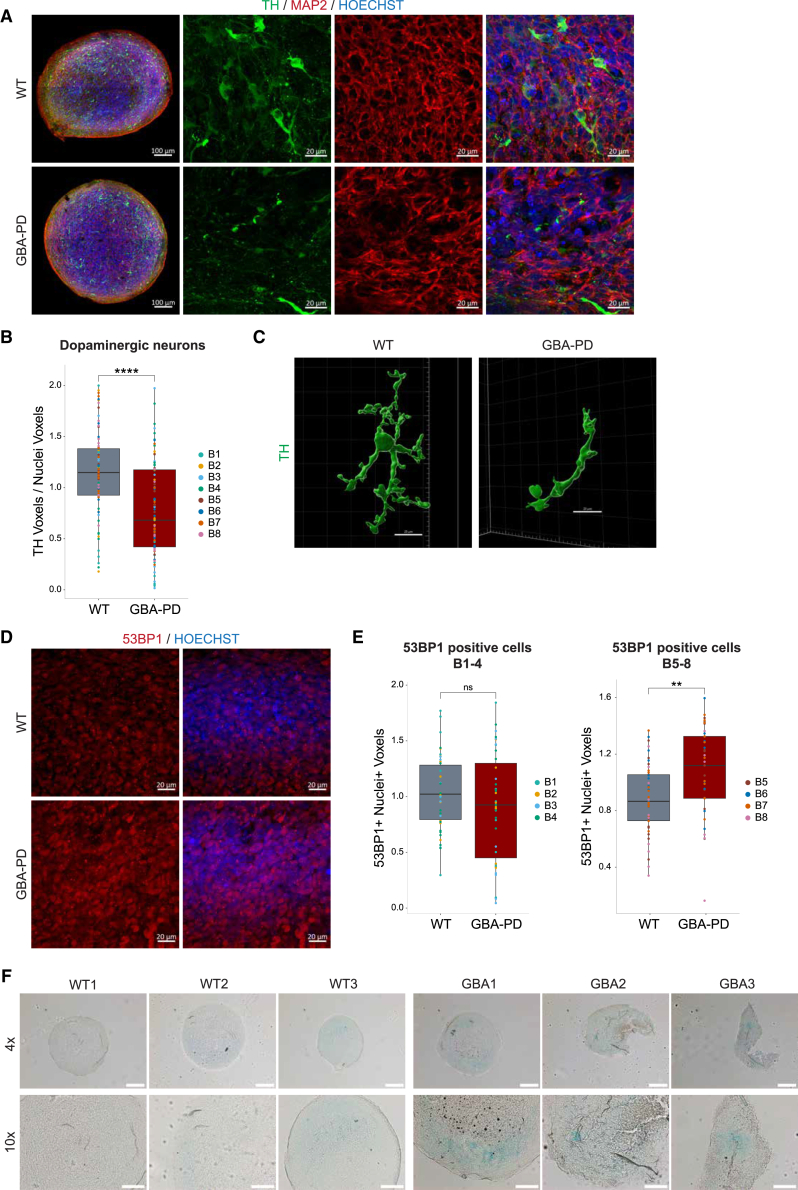
Reproducible dopaminergic neuron and senescence phenotypes at the protein level in midbrain organoids Midbrain organoids were generated at early or late passage in four independent batches, and whole-mount immunofluorescence and β-galactosidase staining were performed. (A) Representative confocal images of TH (green), MAP2 (red), and nuclei (blue) of midbrain organoid section staining at day 30 (scale bars, 100 μm, 20×; 50 μm, 63×). (B) High-content automated image analysis of immunofluorescence staining of TH+ dopaminergic neurons in midbrain organoids normalized by total nuclei. Data are shown as boxplots and represent a summary of eight independent batches normalized to the mean of the controls per batch ±SD. Wilcoxon t test; ∗∗∗∗*p* < 0.0001. (C) 3D reconstruction with IMARIS software showing the dopaminergic neuron (TH) in the healthy control (WT) and GBA-PD cell line (scale bars, 20 μm, 63×). (D) Representative confocal images of 53BP1 (red) and nuclei (blue) in whole-mount staining of midbrain organoids at day 30 (scale bars, 20 μm, 63×). (E) High-content automated image analysis of immunofluorescence staining of 53BP1 (53BP1+) and Nuclei (Nuclei+) double-positive cells in midbrain organoids. Data are shown as boxplots and represent early-passage (B1–4) or late-passage (B5–8) batches normalized to the mean of the controls per batch ±SD. Wilcoxon t test; ∗∗*p* < 0.01. (F) Senescence-associated β-galactosidase staining (blue) of midbrain organoids (scale bars, 200 μm, 4×; 100 μm, 10×).

Our transcriptomic analysis identified a distinct disease signature in GBA-PD, characterized by significant dysregulation of genes associated with pathways related to cellular senescence and apoptosis, as well as differences in synaptic and nervous system development. To validate these transcriptomic results and confirm phenotype reproducibility at the protein level, we performed whole-mount staining of midbrain organoids. We used the dopaminergic neuron marker TH (tyrosine hydroxylase), the neuronal marker MAP2 (microtubule-associated protein 2), and the DNA damage/senescence marker 53BP1 (p53-binding protein 1) to additionally assess dopaminergic neurons and the senescence phenotypes at the protein level. We observed that GBA-PD midbrain organoids exhibit a significantly reduced number of TH-positive dopaminergic neurons compared to WT midbrain organoids consistent with results previously reported^10^^,^^11^ (Figures 4A and 4B). Similarly, 3D reconstruction of dopaminergic neurons showed that neurites in patient-specific organoids are shorter and less ramified compared to WT (Figure 4C). Assessing the TH transcript counts in the transcriptomic data, we observed a significant decrease in the GBA-PD cell lines, supporting the immunofluorescence findings (Figure S3A). The log2 FC of the TH expression in GBA-PD vs. WT appeared more heterogeneous in late-passage batches (B5–8) compared to the early-passage batches (B1–4) (Figure S3B), confirming that early-passage batches are more reproducible at day 30 of organoid culture. Additionally, we observed that increasing the sample size through individual batches plays a critical role in detecting even the most important phenotypes (Figures S3C and S3D).

Analysis of the DNA damage/senescence marker 53BP1 showed that the senescence phenotype at the protein level appears in the late-passage batches (B5–8), while the transcriptomic signature indicated a significant senescence-associated phenotype already in the early-passage batches (B1–4) (Figures 4D and 4E; Figures S4A and S4B). To further confirm the senescence phenotype in GBA-PD samples, we stained the organoids with β-galactosidase, a well-established marker for detecting cellular senescence.^12^ We qualitatively observed a more intense β-galactosidase signal in GBA-PD midbrain organoids (Figure 4F), providing additional evidence of increased senescence in midbrain organoids derived from patients with PD.^10^

### GBA-PD midbrain organoids demonstrate a reproducible metabolic profile

**Figure 5:**
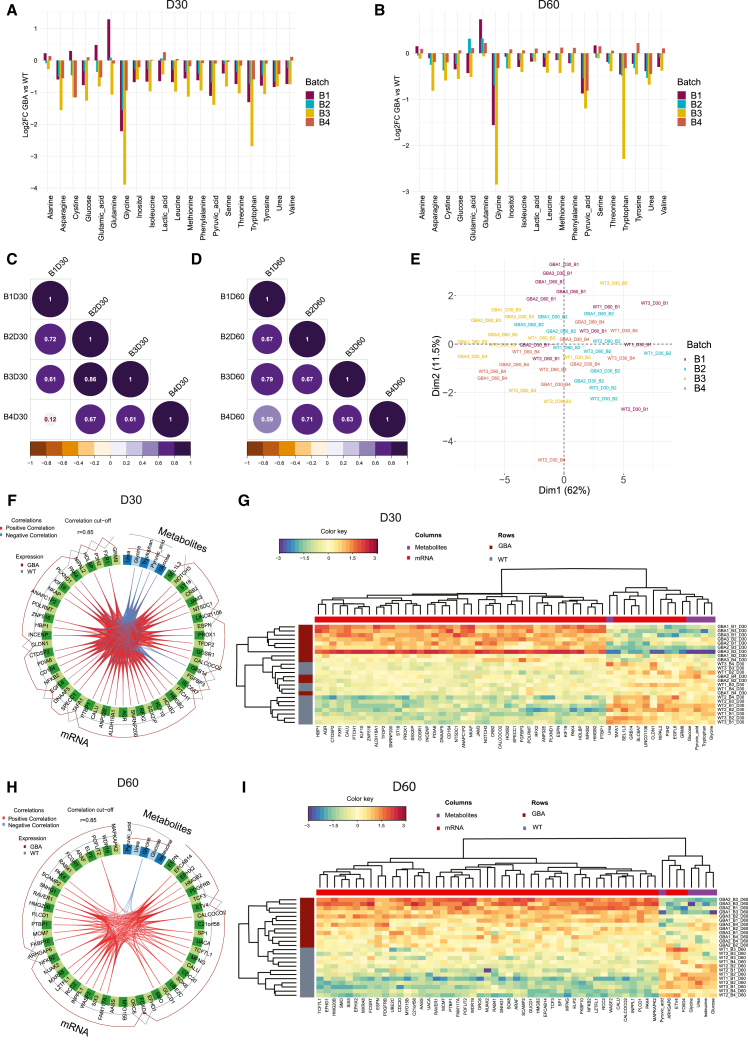
Midbrain organoids from day 30 and day 60 show a reproducible metabolic profile (A) Metabolite abundance difference in GBA-PD samples presented as log2 fold change (FC) compared to healthy control (WT) samples across four batches at day 30 of organoid culture. (B) Metabolite abundance difference in GBA-PD samples presented as log2 FC compared to healthy control (WT) samples across four batches at day 60 of organoid culture. (C) Pearson correlation of log2 FC for all metabolites across four batches at day 30 of organoid culture. (D) Pearson correlation of log2 FC for all metabolites across four batches at day 60 of organoid culture. (E) PCA of metabolomics data. (F) Circos plot showing the correlation between metabolomics and transcriptomics features contributing to the variation of the component 1 at day 30 of organoid culture. Correlation threshold: r = 0.9. (G) Unsupervised hierarchical clustering of the top discriminant metabolomics and transcriptomics features between the GBA-PD and healthy control (WT) samples at day 30 of organoid culture. (H) Circos plot showing the correlation between metabolomics and transcriptomics features contributing to the variation of component 1 at day 60 of organoid culture. Correlation threshold: r = 0.84. (I) Unsupervised hierarchical clustering of the top discriminant metabolomics and transcriptomics features between the GBA-PD and healthy control (WT) samples at day 30 of organoid culture.

After confirming the phenotype reproducibility at the transcriptomic and protein levels, we wanted to assess how reproducible the GBA-PD metabolic signature was. Metabolism is highly dynamic, and therefore metabolite levels may exhibit greater variability between organoid batches compared to changes in transcript or protein abundance. Nevertheless, our data showed that the majority of measured metabolite expression patterns were consistent across four batches at both time points (Figures 5A and 5B). As an exception, we identified B4 to display the most distinct metabolite abundance profile compared to the other three batches at day 30 of organoid culture. Accordingly, correlation analysis showed a significant correlation of metabolite FCs (GBA-PD vs. WT) for B1, B2, and B3 (0.61–0.86), while B4 demonstrated a low correlation with any of these batches (0.12) (Figure 5C). However, at the later time point (day 60), all four batches showed a significant correlation (0.59–0.79), suggesting that the differences in B4 at day 30 might be due to an organoid differentiation path that transiently diverges from the other batches, but these differences even out in more mature organoid cultures (Figure 5D). Moreover, PCA analysis demonstrated that the metabolic profiles of WT and GBA-PD midbrain organoids are not influenced by batch effects but are instead driven by sample and disease state, further supporting the reliability and reproducibility of the data (Figure 5E).

In addition, we performed an integrative analysis between the metabolomics and transcriptomics datasets to identify the key molecular interactions across different omics levels at different organoid culturing stages (day 30 and day 60). Importantly, this analysis confirmed earlier findings, demonstrating that the top transcripts and metabolites more effectively distinguish samples by condition rather than by batch (Figure S5). We observed that pyruvic acid, urea, glycine, and glucose were reappearing among the top five metabolites separating the WT and GBA-PD samples at both time points (Figures 5A–5I). Moreover, at both time points, the change in glycine abundance between conditions was negatively correlated with a subset of the top 50 genes, suggesting that glycine metabolism and its transcriptional regulation play a substantial role in GBA-PD (Figures 5F and 5H). Aligned with overall metabolism adaptations usually observed during aging and the metabolic alterations associated with GBA mutation-driven dysregulation and senescence, we found improved sample separation based on top transcriptomic and metabolomic features at day 60 of organoid culture (Figures 5G and 5I). This finding suggests that the metabolic phenotype of GBA-PD intensifies over time (Figures 5G and 5I).

Altogether, these data suggest that even highly dynamic metabolic changes are reproducible in midbrain organoid models and therefore can reliably capture critical molecular interactions driving GBA-PD metabolic dysregulation.

### Sample estimation using power analysis for iPSC-derived models

**Figure 6:**
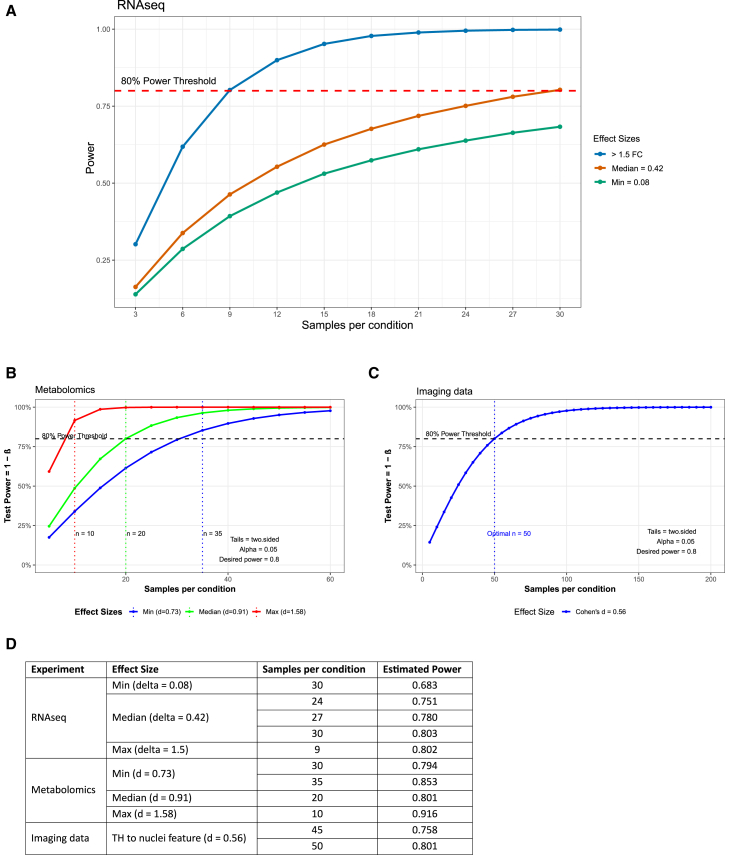
Sample estimation using power analysis for three data types (RNA-seq, metabolomics, and imaging) Each panel plots statistical power (1-β) against sample size per group at α = 0.05, highlighting the sample sizes needed to reach the 80% power threshold (dashed line) for different effect sizes determined from this pilot study in each data type. (A) RNA-seq shows effect sizes of >1.5-fold (blue), median = 0.42 (orange), and min = 0.08 (green). (B) Metabolomics compares min (*d* = 0.73), median (*d* = 0.91), and max (*d* = 1.58) effect sizes; vertical lines mark the number of replicates at which each effect achieves 80% power. (C) Imaging data illustrate a single effect size (Cohen’s *d* = 0.56), requiring ∼*n* = 50 per group to reach 80% power. (D) Table summarizing the power analysis results showing the effect size, the sample size per group, and the estimated power for each experiment performed in this study (RNA-seq, metabolomics, and imaging data).

A considerable limitation of studies using iPSC-derived models is the availability of cell lines, particularly when it comes to patient-specific lines with a particular mutation. This leads to underpowered studies, increasing the risk of reporting false-negative results (type II error).^13^ We observed exactly this in our example study on TH gene expression presented here, where a single batch with three samples per group failed to capture the true differences in TH expression levels between the control and GBA-PD samples. Therefore, using independent experimental batches can be used to enhance the experimental throughput by increasing sample sizes. Thus, in addition to assessing the reproducibility of the midbrain organoid model, we wanted to estimate the required sample size for RNA-seq, metabolomics, and imaging experiments to reach an optimal statistical power of 80%, considering the presented data as pilot experiments for future experimental designs using organoid models. 80% statistical power is, by convention, the target power of a study to reduce the probability of rejecting a false null hypothesis.^14^ For RNA-seq datasets, we estimated how many samples per group are required to reach optimal statistical power based on the observed effect size as log2 FC between the significant DEGs in this study. While the absolute values of log2 FC ranged from 0.08 (min) to 8.21 (max), the median was 0.42, indicating that for most of the DEGs, the difference between the two groups is relatively small and 30 samples per group would be required to detect true differences in the gene expression (Figures 6A and 6D). This means that for the most differentially expressed transcripts, 10 batches must be generated to detect true significant differences between the groups if we have 3 cell lines from each group or condition available. For a larger effect size between groups (>1.5 log2 FC), the sample size required for optimal power of 80% decreases to 9 samples per group. Consequently, three independent rounds of organoid generation are required to detect significantly DEGs with an expected expression difference greater than 1.5-fold when three cell lines per group or condition are used. In the metabolomics dataset, we estimated the effect size as Cohen’s *d* of each significantly differentially abundant metabolite. Median *d* was large (0.91), which would require 20 samples per sample group to reach optimal statistical power in the analysis (Figures 6B and 6D). The maximum *d* was 1.58, decreasing the required sample number to ten, while the smallest *d* was 0.73, increasing the required sample number to 35 per group (Figures 6B and 6D). In the imaging dataset, we estimated the required sample size based on the values of TH normalized by nuclei, as this is the expected imaging feature to be significantly different between the healthy control and GBA-PD samples. With the calculated effect size d = 0.56, 50 samples per group would be necessary to reach 80% statistical power (Figures 6C and 6D). In organoid studies, immunofluorescence staining is performed on organoid sections. Unlike RNA-seq or metabolomics experiments, where organoids are pooled into a single sample represented by one data point in the analysis, in imaging experiments, we consider each individual organoid as a biological replicate. This increases the sample size, which allows for optimal statistical power considering the rather low expected effect size between the sample groups in imaging.

For instance, in this pilot study, the use of three cell lines and eight batches per group increases the sample size to 24 per group. Similar approaches can be used to achieve the optimal sample size, considering the specific study objective and the standards required.^15^

This analysis highlights the importance of incorporating multiple batches to achieve statistically robust and reproducible results, when the availability of biological replicates (cell lines) is limited. However, increasing the number of cell lines is always preferred over adding more batches, as it results in more generalizable and translatable findings.

## Discussion

Our study establishes midbrain organoids as a reproducible *in vitro* model to study PD. By generating multiple independent organoid batches and examining both early and late passages, as well as different time points, we address a key challenge in the field: ensuring reproducibility and consistency in complex 3D culture systems.

Our findings reveal that, despite the inherent complexity of organoid models, batch effects can be successfully minimized. PVCA underscores that donor-related factors, disease state, and sex are prominent drivers of transcriptional variability, while passage of the NESCs emerges as a technical parameter contributing to transcriptional shifts. Notably, early-passage cultures consistently yield a more robust and stable disease-specific transcriptomic signature compared to late-passage organoids, which nonetheless still maintain core disease-related features. This signature, associated with TH-positive dopaminergic neurons loss and cellular senescence, aligns with previously reported phenotypes in GBA-PD models and reflects key pathogenic mechanisms implicated in disease progression.^10^^,^^11^

Crucially, we validated these transcriptomic differences at the protein and metabolic levels. Changes in dopaminergic neuron numbers, along with increased senescence-associated markers, were consistent with our gene expression data. Furthermore, the metabolic profiles were largely stable and reproducible across independent batches of organoid generation, particularly as the organoids matured. This evidence supports the notion that midbrain organoids can recapitulate key aspects of PD pathology reproducibly along multi-level analysis.

Although this study focused on PD patient midbrain organoid samples carrying the GBA-N370S genetic variant, we believe that the number of samples from independent organoid generation batches was sufficient to assess the overall variation in the organoid data. Thus, our conclusions on organoid reproducibility can likely be generalized to other organoid experiments involving two or more independent sample groups. Furthermore, our analysis incorporated sample size estimation to achieve optimal statistical power across bulk RNA-seq, immunofluorescence high-content imaging, and metabolomics experiments, offering guidelines for designing future organoid studies to ensure result accuracy and minimize false-positive results. It is important to note that midbrain organoids represent a guided brain organoid model, where variability between individual organoids and batches is expected to be lower compared to unguided brain organoid protocols. In such unguided models, larger sample sizes may be necessary to reliably detect true differences between sample groups.

While including batches can enhance the statistical power of a study, they should not be used to artificially inflate power. Instead, batches should serve as an additional replicate alongside multiple distinct cell lines (if available) or used as a strategy to help overcome the common challenge of limited cell line availability (e.g., in the case of rare mutations).

In conclusion, this study provides strong evidence that patient-derived midbrain organoids are not only an accurate model of GBA-PD pathology but are also a robust and reproducible experimental system. By highlighting and controlling critical sources of variability, these models can build a solid foundation for future work aimed at unraveling disease mechanisms and accelerating the development of personalized treatments for PD.

### Limitations of the study

Despite the strengths of our study, several limitations should be acknowledged. While we used multiple organoid batches and time points, our analysis focused exclusively on midbrain organoids derived from patients with PD carrying the GBA-N370S mutation, which may limit generalizability to other PD genetic backgrounds. The relatively small number of distinct donor lines, though sufficient for detecting robust transcriptomic and phenotypic changes, may not fully capture the spectrum of patient heterogeneity. A key consideration is that NESC passage number introduced greater variability than organoid batch, which could affect reproducibility if not carefully controlled. Early-passage organoids showed more consistent phenotypes, making them more suitable for disease modeling. In contrast, late-passage organoids exhibited greater variability but also signs of transcriptional aging. This suggests their potential for modeling late-stage phenotypes, though early and late passages should be analyzed separately to preserve reproducibility. Although we implemented power calculations, limited cell line availability, especially for rare mutations, remains a challenge for study robustness. To further evaluate the generalizability of our findings, future studies should include organoids derived from patients carrying other GBA variants or additional PD-linked mutations (e.g., LRRK2, SNCA, and Miro1) to assess reproducibility across diverse genetic backgrounds. A more balanced inclusion of female and male donor lines, combined with minimized technical variability such as consistent passage number, would also enable systematic investigation of sex-specific transcriptomic effects in disease modeling. Together, these approaches would help validate midbrain organoids as a consistent and versatile *in vitro* model for studying PD.

## Resource availability

### Lead contact

Further information and requests for resources should be directed to and will be fulfilled by the lead contact, Jens C. Schwamborn (jens.schwamborn@uni.lu).

### Materials availability

This study did not generate new unique reagents.

### Data and code availability

• All original and processed data as well as scripts that support the findings of this study are public available at https://doi.org/10.17881/4n7e-vc76.
• Gene expression datasets can be accessed on Gene Expression Omnibus under the accession codes GSE287566 and GSE269316.
• All scripts used to obtain, analyze, and plot the data are available at https://gitlab.com/uniluxembourg/lcsb/developmental-and-cellular-biology/reproducibility_2025.
• Any additional information required to reanalyze the data reported in this article is available from the lead contact upon request.

## Acknowledgments

We thank the LCSB Metabolomics and Lipidomics Platform for their service. We acknowledge Novogene for RNA sequencing. We would like to thank the LCSB Bioimaging Platform for supporting high-content imaging and image analysis workflow. We would also like to thank Dr. Sònia Sabaté Soler for the help with the 3D reconstruction using the IMARIS software and Dr. Matthieu Gobin for his critical contributions to the manuscript.

This project has received funding from the National Centre of Excellence in Research on Parkinson’s Disease (NCER-PD), which is funded by the Luxembourg National Research Fund (10.13039/501100001866FNR) (FNR/NCER13/BM/11264123). Further, FNR funding as part of the i2TRON Doctoral Training Unit (PRIDE19/14254520) and the CORE program (CORE 22_BM_17193204_MidStriPD) is acknowledged.

This research was funded in whole by the 10.13039/501100001866FNR Luxembourg. For the purpose of Open Access, the author has applied a CC BY public copyright license to any Author Accepted Manuscript (AAM) version arising from this submission.

## Author contributions

E.Z. and A.Z. conceived, designed, and collected data. E.Z., H.A.S., and A.Z. performed data analysis and interpretation of results. M.T. contributed with experiments. E.Z., H.A.S., and A.Z. wrote the original manuscript. The work was supervised by J.C.S. S.L.N. and J.C.S. revised and edited the manuscript.

## Declaration of interests

J.C.S. declare no competing non-financial interests but declare competing financial interests as cofounders and shareholders of OrganoTherapeutics société à responsabilité limitée.

## STAR★Methods

### Key resources table

**Table:** 

REAGENT or RESOURCE	SOURCE	IDENTIFIER
**Antibodies**		

Chicken polyclonal anti-TH	Abcam	Cat#ab76442; RRID:AB_1524535
Rabbit polyclonal anti-TH	Abcam	Cat# ab112; RRID:AB_297840
Mouse monoclonal anti-TUJ1	BioLegend	Cat#801201; RRID:AB_2313773
Rabbit polyclonal anti-53BP1	Novus Biologicals	Cat# NB100-304; RRID:AB_10003037
Chicken polyclonal anti-MAP2	Abcam	Cat# ab92434; RRID:AB_2138147
Alexa Fluor® 488 AffiniPure® Donkey Anti-Chicken IgY (IgG) (H + L)	Jackson Immunoresearch	Cat#703-545-155; RRID:AB_2340375
Alexa Fluor® 647 AffiniPure® Donkey Anti-Chicken IgY (IgG) (H + L)	Jackson Immunoresearch	Cat#703-605-155; RRID:AB_2340379
Donkey anti-Rabbit IgG (H + L) Highly Cross-Adsorbed Secondary Antibody, Alexa Fluor™ 488	Invitrogen	Cat# A21206; RRID:AB_2535792
Donkey anti-Rabbit IgG (H + L) Highly Cross-Adsorbed Secondary Antibody, Alexa Fluor™ 568	Invitrogen	Cat# A-10042; RRID:AB_2534017
Donkey anti-Mouse IgG (H + L) Highly Cross-Adsorbed Secondary Antibody, Alexa Fluor™ 647	Invitrogen	Cat# A-31571; RRID:AB_162542

**Chemicals, peptides, and recombinant proteins**		

Geltrex	Gibco	A1413302
DMEM/F-12, no glutamine	Gibco	21331046
Neurobasal Medium	Gibco	21103049
GlutaMAX	Gibco	35050061
Penicillin-Streptomycin, liquid	Gibco	15140122
B-27 Supplement Minus Vitamin A (50×), Liquid	Gibco	12587001
*N*-2 Supplement (100×)	Gibco	17502001
L-Ascorbic acid	Sigma-Aldrich	A4544
CHIR99021	Axon Medchem BV	AXON1386
DONKEY SERUM Purmorphamin	Enzo Life Science	ALX-420-045
Accutase	Sigma-Aldrich	A6964
dbcAMP	STEMCELL Technologies	100–0244
hBDNF	PeproTech EC Ltd.	450–02
hGDNF	PeproTech EC Ltd.	450–10
TGF-β3	PeproTech EC Ltd.	100-36E
Paraformaldehyde	Sigma-Aldrich	P6148
Hoechst33342	Invitrogen	H21492
Triton X-100	Carl Roth	3051.3
Bovine Serum Albumin	Sigma-Aldrich	A4503
Donkey Serum	Sigma Aldrich	D9663
Sodium Azide	Carl Roth	K305.1
Fluoromount-G	Southern Biotech	SOUT0100-01

**Critical commercial assays**		

LookOut® Mycoplasma PCR Detection Kit	Sigma-Aldrich	MP0035-1 KT
Senescence Detection Kit	Abcam	ab65351
RNeasy Mini Kit	Qiagen	74106

**Deposited data**		

Bulk RNA sequencing dataset 1	This paper	GSE287566
Bulk RNA sequencing dataset 2	This paper	GSE269316
Figures and Source codes	This paper	https://doi.org/10.17881/4n7e-vc76

**Experimental models: Cell lines**		

iPSC line WT1	IBBL/Max Planck Institute	N/A
iPSC line WT2	IBBL/Max Planck Institute	N/A
iPSC line WT3	Coriell Institute	GM23338
iPSC line GBA1	European Bank for induced pluripotent Stem Cells	UOXFi001-B
iPSC line GBA2	University College London	N/A
iPSC line GBA3	Coriell Institute	ND31630

**Software and algorithms**		

IMARIS	Bitplane software	Version 9.9.1.; RRID:SCR_007370
Fiji	https://fiji.sc/	RRID:SCR_002285
MATLAB	Matchworks, Inc.	R2021a; RRID:SCR_001622
Galaxy	Galaxy server	Version 23.2.rc1
HISAT2	https://daehwankimlab.github.io/hisat2/	RRID:SCR_015530
featureCounts	http://bioinf.wehi.edu.au/featureCounts/	RRID:SCR_001905
R Project for Statistical Computing	https://www.r-project.org/	Version 4.4.2.; RRID:SCR_001905
DESeq2 R package	https://bioconductor.org/packages/release/bioc/html/DESeq2.html	Version 1.42.1.; RRID:SCR_015687
dplyr R package	https://dplyr.tidyverse.org/	Version 1.1.4.; RRID:SCR_016708
PVCA R package	https://www.bioconductor.org/packages/release/bioc/html/pvca.html	Version 1.42.0.; RRID:SCR_001356
mixOmics R package	http://mixomics.org/access/	Version 6.22.0.; RRID:SCR_016889
Circos R package	http://circos.ca/	Version 0.69–9.; RRID:SCR_011798
PROPER R package	https://bioconductor.org/packages/release/bioc/html/PROPER.html	Version 1.30.0.
pwr R package	https://cran.r-project.org/web/packages/pwr/index.html	Version 1.3.0.; RRID:SCR_025480
effsize R package	https://cran.r-project.org/web/packages/effsize/effsize.pdf	Version 0.8.1.
EnrichR tool	https://maayanlab.cloud/Enrichr/	RRID:SCR_001575
Illustrator	Adobe Systems, Inc.	Version 29.5.1.; RRID:SCR_010279
Photoshop	Adobe Systems, Inc.	Version 26.6.1.; RRID:SCR_014199

### Experimental model and subject details

#### Ethical approval

The work with iPSCs has been approved by the Ethics Review Panel (ERP) of the University of Luxembourg and the national Luxembourgish Research Ethics Committee (CNER, Comité National d'Ethique de Recherche) under CNER No. 201901/01 (ivPD) and No. 202406/03 (AdvanceOrg).

#### Cell lines

The patient GBA1 cell line was obtained from the European Bank for induced pluripotent Stem Cells (EBiSC), the patient GBA2 cell line was provided by the University College London and the patient GBA3 cell line from the Coriell Institute. The healthy control 1 and 2 were generated at the Max Planck Institute and healthy control 3 was provided by the Coriell Institute (see Table S1).

#### Midbrain organoid culture

One iPSC line from each donor was used, which was derived into NESCs as described by Reinhardt et al.^16^ Midbrain organoids were generated from NESCs according to a protocol published by Monzel et al.^4^ and Nickels et al.^5^ until day 30 or day 60 of organoid culture depending on the experiment. NESCs were cultured in N2B27 maintenance media in 6-well Geltrex (Gibco, cat. no. A1413302) precoated plates. The N2B27 base media consists of DMEM/F-12 (Gibco cat. no. 21331046) and Neurobasal (Gibco, cat. no. 21103049) in a 50:50 ratio and is supplemented with 1% GlutaMAX (Gibco, cat. no. 35050061), 1% penicillin/streptomycin (Gibco, cat. no. 15140122), 1% B27 supplement w/o Vitamin A (Gibco, cat. no. 12587001) and 2% N2 supplement (Gibco, cat. no. 17502001). For the maintenance of NESCs, the N2B27 base media was supplemented with 150 μM ascorbic acid (Sigma-Aldrich, cat. no. A4544), 3 μM CHIR-99021 (Axon Medchem BV, cat. no. AXON1386), and 0.75 μM purmorphamine (Enzo Life Science, cat. no. ALX-420-045). The NESC maintenance media was exchanged every second day. For the generation of midbrain organoids, NESCs were detached at 80% confluence with Accutase (Sigma-Aldrich, cat. no. A6964). Trypan Blue was used to count the number of viable cells. 9,000 live cells were seeded into each well of a 96-well ultra-low attachment plate (faCellitate, cat. no. F202003) in 150 μL of NESC maintenance media to initiate spheroid formation, marking day 0 of organoid culture. On the 2^nd^ day of midbrain organoid culture, the maintenance media was changed to patterning media, where N2B27 base media was supplemented with 200 μM ascorbic acid (Sigma-Aldrich, cat. no. A4544), 500 μM dbcAMP (STEMCELL Technologies, cat. no. 100–0244), 10 ng/mL hBDNF (PeproTech EC Ltd., cat. no. 450-02), 10 ng/mL hGDNF (PeproTech EC Ltd., cat. no. 450-10), 1 ng/mL TGF-β3 (PeproTech EC Ltd., cat. no. 100-36E) and 1 μM purmorphamine (Enzo Life Science, cat. no. ALX-420-045). The next media change with the pattering media was done on the 5^th^ day of organoid culture. On the 8^th^ day of organoid culture, the patterning media was replaced by the differentiation media, which excluded PMA from the patterning media composition. Further media changes were done every 3–4 days until sample collection. NESC and midbrain organoid cultures were regularly (once per month) tested for mycoplasma contamination using LookOut Mycoplasma PCR Detection Kit (Sigma-Aldrich, cat. no. MP0035-1 KT).

### Method details

#### Whole mount staining

Midbrain organoids were collected at day 30, fixed with 4% paraformaldehyde (PFA) (Sigma-Aldrich, cat. no. P6148) overnight at 4 °C and washed 3× with PBS for 10 min. The whole organoids were permeabilized and blocked with 1% Triton X-100 (Carl Roth, cat. no. 3051.3) and 10% donkey serum (Sigma-Aldrich, cat. no D9663) in PBS for 24 h at room temperature (RT) and washed with 0.01% Triton X-100 in PBS for 30 min at RT on an orbital shaker. The organoids were incubated for 4 days at 4 °C on an orbital shaker with the primary antibodies (Table S2) diluted in 0.5% Triton X-100 and 3% donkey serum in PBS. They were washed 3× with PBS for 1 h before incubation for 2 days at 4 °C on an orbital shaker with secondary antibodies (Table S2) diluted in 0.5% Triton X-100 and 3% donkey serum in PBS. The whole organoids were washed 3× with 0.05% Triton X-100 in PBS and once with Milli-Q water for 5 min at RT before mounting them with Fluoromount-G mounting medium (Southern Biotech, cat. no. SOUT0100-01).

#### Immunofluorescence staining of midbrain organoid sections

Midbrain organoids were collected at day 30, fixed with 4% paraformaldehyde (PFA) overnight at 4 °C and washed 3× with PBS for 10 min. Four midbrain organoids per cell line were embedded in 3% low-melting point agarose (Biozym, cat. no. 840100) and the solid agarose block with the assembloid was sectioned at 60 μm using the vibrating blade microtome (Leica VT1000s, RRID:SCR_016495). The sections were permeabilized for 30 min in 0.5% Triton X-100 at RT, followed by one quick wash with 0.01% Triton X-100 in PBS. The sections were then blocked for 2h at RT with blocking buffer containing 2.5% BSA (Sigma-Aldrich, cat. no. A4503), 2.5% donkey serum, 0.01% Triton X-100 and 0.1% sodium azide (Carl Roth, cat. no. K305.1) in PBS. Primary antibody (Table S2) was diluted in blocking buffer and the sections were incubated with the primary antibody dilutions for 48h at 4°C on an orbital shaker. Incubation with secondary antibodies and mounting of the sections were performed as previously described.^5^

#### Image 3D reconstruction

The 3D structure of the dopaminergic neuron was reconstructed from whole mount stainings using IMARIS software (version 9.9.1., (Bitplane, RRID:SCR_007370). z stack planes were used to visualise the fragmentation of dopaminergic neurons positive to tyrosine hydroxylase (TH) in GBA-PD compared to hearty control (WT) midbrain organoids.

#### β-galactosidase staining

60 μm sections from midbrain organoids were used in the β-galactosidase staining, using the Senescence Detection Kit (Abcam, cat.no. ab65351). Two sections from one midbrain organoid per cell line were used. Images were acquired at 4× and 10× on an Olympus IX83 Automated Fluorescence Microscope (RRID:SCR_020344) for qualitative images and enhanced using Fiji software (RRID:SCR_002285) to account solely for differences in the background levels of light.

#### Image acquisition and analysis

For high-content imaging, mounted organoids were scanned using the Yokogawa CellVoyager CV8000 microscope (RRID:SCR_023270). A 4× pre-scan in the 405 channel identified organoid-containing wells, enabling the creation of masks to outline organoids. These masks guided the selection of the field for imaging at different wavelengths with a 20× objective. For all stainings, three to four organoids per condition and four batches were analyzed, with details provided in figure legends. Qualitative images were captured using a Zeiss LSM 710 Confocal Inverted Microscope (RRID:SCR_018063) with 20×, or 60× objectives.

Immunofluorescence images of the whole mount organoids from the Yokogawa microscope were analyzed in MATLAB (2021a, Mathworks, RRID:SCR_001622) using a custom image-analysis algorithm as described by Bolognin et al.^17^ The algorithm merges overlapping sections into mosaic images, smoothes and combines color channels and removes small objects. Masks were created and refined for each marker based on pixel intensity to quantify marker areas in 3D space (voxels). Representative images were edited with Adobe Illustrator (version 29.5.1., RRID:SCR_010279) and Adobe Photoshop (version 26.6.1, RRID:SCR_014199) for visualization purposes.

#### RNA extraction, library preparation and sequencing

Total RNA was extracted from each organoid generation batch, with 15–20 midbrain organoids pooled per batch at either day 30 or day 60. RNA isolation was done using the RNeasy Mini Kit (Qiagen, cat. no. 74106) following the manufacturer’s protocol. Messenger RNA was purified from total RNA using poly-T oligo-attached magnetic beads. After fragmentation, the first strand cDNA was synthesized using random hexamer primers, followed by the second strand cDNA synthesis using either dUTP for directional library or dTTP for non-directional library. Library preparations were sequenced on an Illumina platform by Novogene’s sequencing service.

#### Transcriptomic analysis

RNA sequencing data were pre-processed on the Galaxy server (version 23.2.rc1) following Galaxy training tutorial.^18^^,^^19^ Reads were mapped to the human reference genome hg38 using the HISAT2 tool (RRID:SCR_015530). Mapped reads were counted using the featureCounts (RRID:SCR_001905) function on the BAM output files of HISAT2. Differential expression analysis was conducted in the R Project for Statistical Computing (version 4.4.2., RRID:SCR_001905) using the software package “DESeq2” (version 1.42.1., RRID:SCR_015687) (Love et al.^20^). *P*-value significance scores for differential expression were adjusted for multiple hypothesis testing according to the Benjamini and Hochberg method.^21^ Pathway enrichment analysis was performed with the EnrichR tool (RRID:SCR_001575)^22^^,^^23^ using the gene-level differential expression results table obtained with the DESeq2. We made use of two Gene-set libraries: the “Reactome Pathways 2024” library^24^ and the “GO Biological process 2023” library.^25^^,^^26^ Euclidean distance analysis was performed in R using base functions for distance computation and the “dplyr” package (version 1.1.4., RRID:SCR_016708) for data manipulation, ensuring precise distance measurements through established statistical functions.^27^^,^^28^ This approach allowed us to quantify the relative positioning of the data points within the multidimensional space defined by the clusters, providing insight into its similarity to each group.

#### Analysis of explained variance

To assess the sources of variability in gene expression data, Principal Variance Component Analysis (PVCA) was performed in R using the “PVCA” package (version 1.42.0., RRID:SCR_001356).^29^ PVCA performs the dual analysis Principal Component Analysis (PCA) and Variance Component Analysis (VCA). This results in a reduction in data dimensionality while retaining most of its variability. A mixed linear model is then applied, treating all factors of interest as random effects, including two-way interaction terms, to estimate and partition the total variance attributed to each term. This method accounts for all defined sources of variability, including both experimental (cell line, passage, batch) and biological (disease status, patient sex) factors. Both, biological and experimental variates were treated as random factors and the variance attributed to other technical variables was assigned to a residual variance category.

#### Transcriptomics and metabolomics integration

RNA sequencing and metabolomics data were integrated using “mixOmics” package (version 6.22.0., RRID:SCR_016889)^30^ in R with DIABLO framework.^31^ The separation between sample groups was assessed using the correlation of the top 50 genes with the top five metabolites from the first component of Partial Least Squares Discriminant Analysis (PLS-DA). Pearson correlation (abs(r) = 0.85) between metabolomics and transcriptomics features was further visualized in a Circos plot (version 0.69–9., RRID:SCR_011798).

#### Sample size estimation using power analysis

For the RNAseq dataset statistical power based on sample size was estimated using “PROPER” package (version 1.30.0.) in R. Library size dispersion and baseline expression values were determined based on the complete RNAseq data (Dataset 1) of midbrain organoid samples from eight independent organoid batches with 48 samples per group (3 cell lines x 2 independent groups (WT and GBA-PD) x 8 independent organoid batches). 20 simulations were run using “DESeq2” (version 1.42.1, RRID:SCR_015687) (Love et al.^20^) as a differential expression analysis method for possible sample sizes considering that we have three independent cell lines per group (WT or GBA-PD), thus sample size can increase by three for each batch (3 cell lines x 2 batches = 6 samples per group). To determine the relevant effect sizes between the two groups, we estimated the median, min and max log2 fold change of significantly differentially expressed genes between GBA and control sample groups. The false discovery rate was set to 5%.

For metabolomics and imaging experiments, sample size estimation was done using “pwr” package (version 1.3.0., RRID:SCR_025480) with a defined significance of alpha = 0.05 and power = 0.8. For both datasets Cohen’s effect size was calculated using the “effsize” package (version 0.8.1.) for significantly different abundant metabolites and TH normalised to nuclei of imaging features respectively. Based on normal data distribution of metabolomics data, we applied pwr.t.test function, while considering potential unequal sample sizes in each group and non-normal data distribution of imaging data, function pwr.2p.test of the pwr package was applied to imaging data.

### Quantification and statistical analysis

#### Statistical analysis

Statistical significance was calculated using Wilcoxon T-test in R Project for Statistical Computing (version 4.4.2., RRID:SCR_001905). Statistically significant results were indicated when *p* values were ∗ <0.05, ∗∗ <0.01, ∗∗∗ <0.001 and ∗∗∗∗ <0.0001. When data was found not significant, it is not specifically stated in the figures and is expressed as ns, not significant. Error bars represent mean +standard deviation (SD). All statistical details of experiments can be found in the Figure legends.
