# Supplementary material for: Reproducibility of PD patient-specific midbrain organoid data for in vitro disease modeling
Source: iScience. 2025 Sep 10;28(10):113541. doi: 10.1016/j.isci.2025.113541 (PMC12494858; doi:10.1016/j.isci.2025.113541)
Supplement: Document S1. Figures S1–S5 and Tables S1 and S2 [file mmc1.pdf]

iScience, Volume *28*

Supplemental information

Reproducibility of PD patient-specific midbrain organoid data for *in vitro* disease modeling

Elisa Zuccoli, Haya Al Sawaf, Mona Tuzza, Sarah L. Nickels, Alise Zagare, and Jens C. Schwamborn

Supplemental Figures

Figure S1

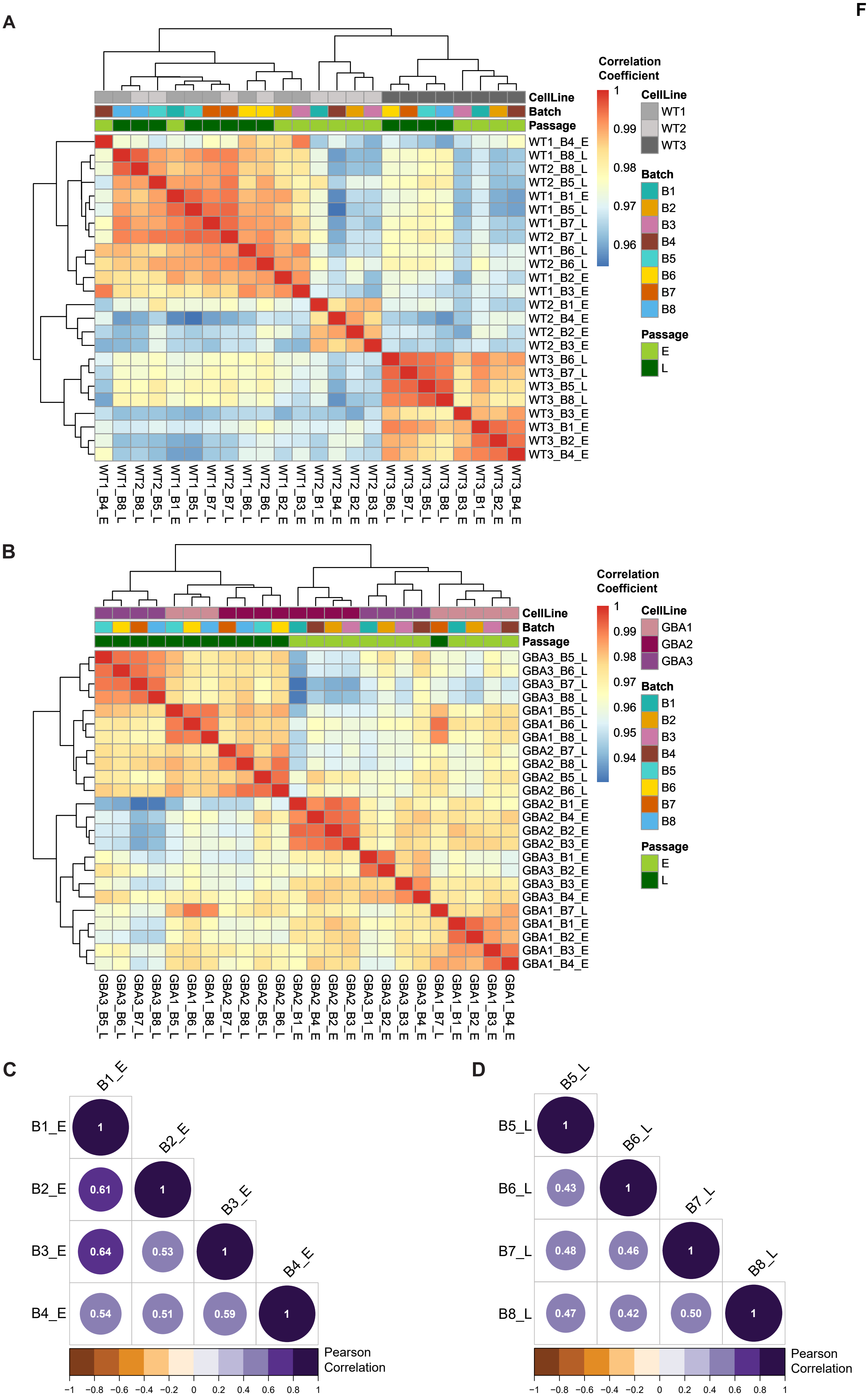

Figure S1: Correlation analysis of healthy control (WT) and GBA-PD samples. Related to Figure 2.

(A) Unsupervised hierarchical clustering plot showing the correlation of healthy control (WT) samples from early and late passage and all eight batches.

(B) Unsupervised hierarchical clustering plot showing the correlation of GBA-PD samples from early and late passage and all eight batches.

(C) Pearson Correlation of log2 fold changes (FC) of all genes between batches derived from early passage NESCs.

(D) Pearson Correlation of log2 fold changes (FC) of all genes between batches derived from late passage NESCs.

Figure S2

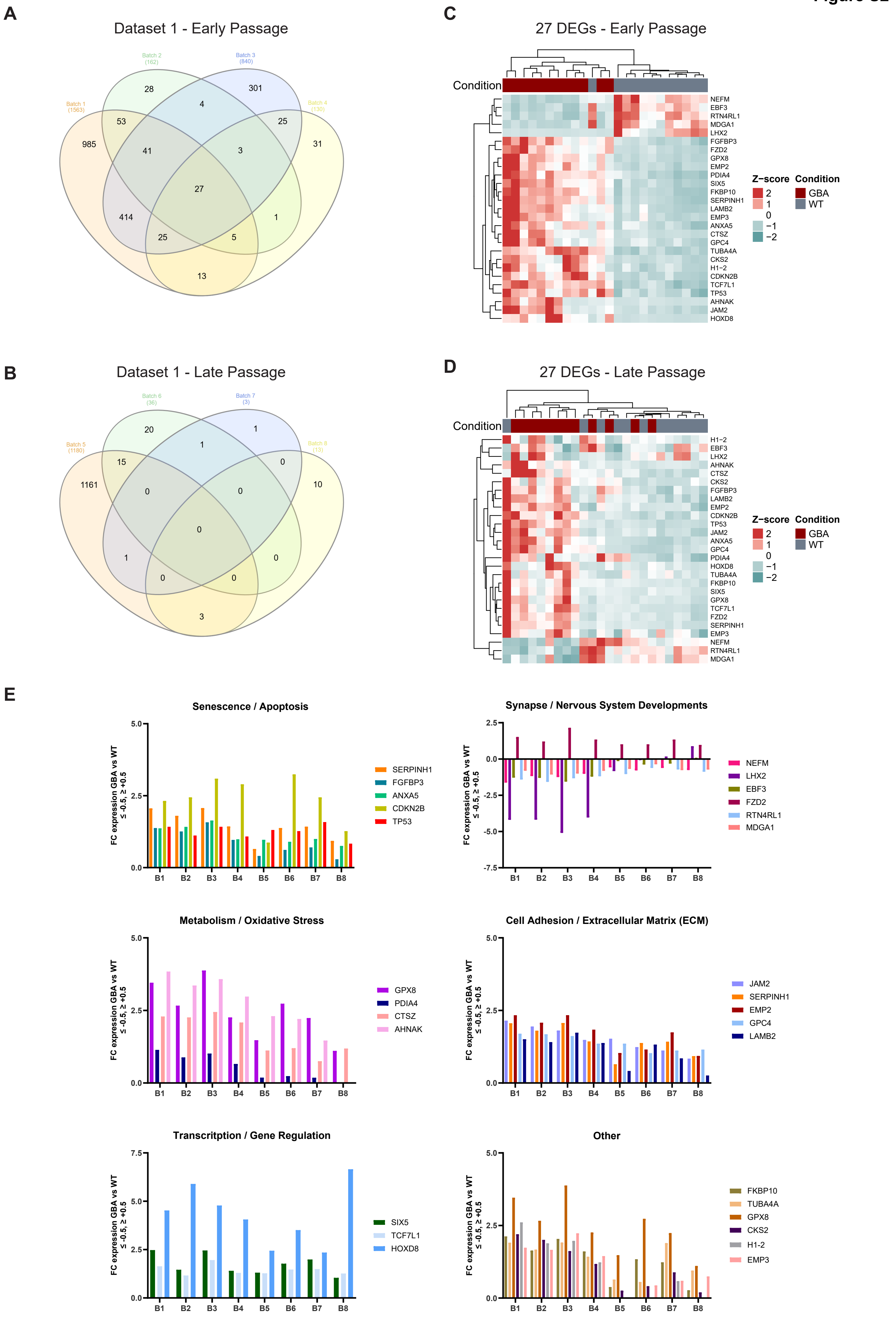
A
Dataset 1 - Early Passage
Batch 1 (1563)
Batch 2 (162)
Batch 3 (840)
Batch 4 (130)
985
28
301
31
53
4
25
41
3
27
414
1
25
5
13
B
Dataset 1 - Late Passage
Batch 5 (1180)
Batch 6 (36)
Batch 7 (3)
Batch 8 (13)
1161
20
1
10
15
1
0
0
0
0
1
0
0
0
3
C
27 DEGs - Early Passage
Condition
NEFM
EBF3
RTN4RL1
MDGA1
LHX2
FGFBP3
FZD2
GPX8
EMP2
PDIA4
SIX5
FKBP10
SERPINH1
LAMB2
EMP3
ANXA5
CTSZ
GPC4
TUBA4A
CKS2
H1−2
CDKN2B
TCF7L1
TP53
AHNAK
JAM2
HOXD8
Z−score
2
1
0
−1
−2
Condition
GBA
WT
D
27 DEGs - Late Passage
Condition
H1−2
EBF3
LHX2
AHNAK
CTSZ
CKS2
FGFBP3
LAMB2
EMP2
CDKN2B
TP53
JAM2
ANXA5
GPC4
PDIA4
HOXD8
TUBA4A
FKBP10
SIX5
GPX8
TCF7L1
FZD2
SERPINH1
EMP3
NEFM
RTN4RL1
MDGA1
Z−score
2
1
0
−1
−2
Condition
GBA
WT
E
Senescence / Apoptosis
FC expression GBA vs WT ≤ -0.5, ≥ +0.5
5.0
2.5
0.0
B1 B2 B3 B4 B5 B6 B7 B8
SERPINH1
FGFBP3
ANXA5
CDKN2B
TP53
Synapse / Nervous System Developments
FC expression GBA vs WT ≤ -0.5, ≥ +0.5
2.5
0.0
-2.5
-5.0
-7.5
B1 B2 B3 B4 B5 B6 B7 B8
NEFM
LHX2
EBF3
FZD2
RTN4RL1
MDGA1
Metabolism / Oxidative Stress
FC expression GBA vs WT ≤ -0.5, ≥ +0.5
5.0
2.5
0.0
B1 B2 B3 B4 B5 B6 B7 B8
GPX8
PDIA4
CTSZ
AHNAK
Cell Adhesion / Extracellular Matrix (ECM)
FC expression GBA vs WT ≤ -0.5, ≥ +0.5
5.0
2.5
0.0
B1 B2 B3 B4 B5 B6 B7 B8
JAM2
SERPINH1
EMP2
GPC4
LAMB2
Transcritption / Gene Regulation
FC expression GBA vs WT ≤ -0.5, ≥ +0.5
7.5
5.0
2.5
0.0
B1 B2 B3 B4 B5 B6 B7 B8
SIX5
TCF7L1
HOXD8
Other
FC expression GBA vs WT ≤ -0.5, ≥ +0.5
5.0
2.5
0.0
B1 B2 B3 B4 B5 B6 B7 B8
FKBP10
TUBA4A
GPX8
CKS2
H1-2
EMP3

Figure S2: Identification of 27 differentially expressed genes (DEGs) in early passage batches and assignment to distinct functional categories. Related to Figure 3.

(A) Venn diagram of DEGs showing the batches derived from early passage NESCs, with 27 genes in common.

(B) Venn diagram of DEGs showing the batches derived from early passage NESCs.

(C) Unsupervised hierarchical clustering of GBA-PD and healthy control (WT) samples based on normalised gene counts of 27 predefined genes for early passage batches.

(D) Unsupervised hierarchical clustering of GBA-PD and healthy control (WT) samples based on normalised gene counts of 27 predefined genes for late passage batches.

(E) Log2 fold changes (FC) of 27 predefined genes divided into distinct functional categories. The genes were assigned to the senescence/apoptosis pathway, synapse and nervous system development, metabolic and oxidative stress processes, cell adhesion and extracellular matrix (ECM) dynamics, and transcription and gene regulation. Each category shows the FC per batch with eight batches in total.

Figure S3

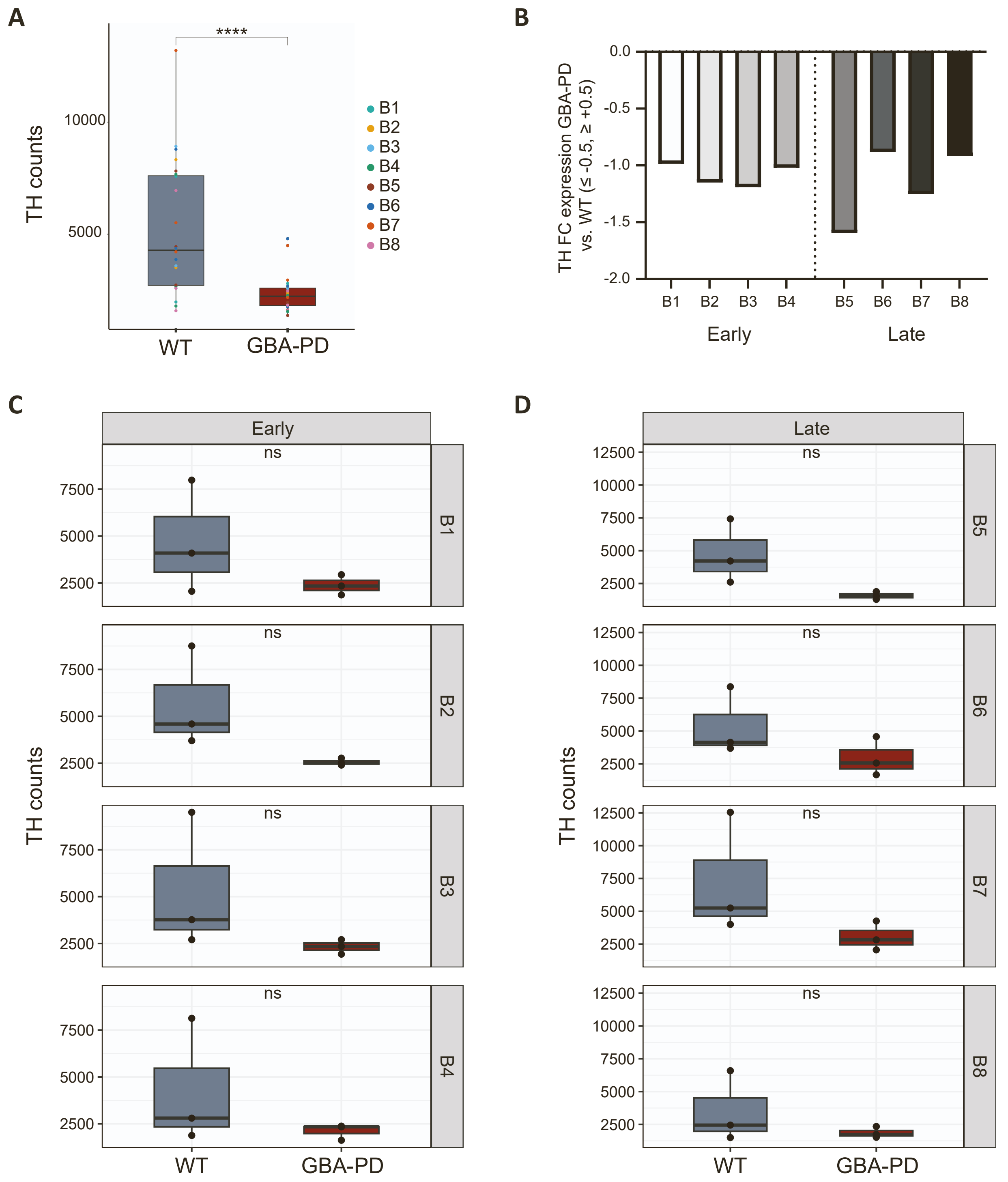
A
TH counts
10000
5000
****
WT
GBA-PD
B1
B2
B3
B4
B5
B6
B7
B8
B
TH FC expression GBA-PD vs. WT (≤ -0.5, ≥ +0.5)
0.0
-0.5
-1.0
-1.5
-2.0
B1 B2 B3 B4 B5 B6 B7 B8
Early
Late
C
Early
ns
TH counts
7500
5000
2500
WT
GBA-PD
D
Late
ns
12500
10000
7500
5000
2500
WT
GBA-PD

Figure S3: Transcriptomic analysis of dopaminergic neuron phenotype supports imaging analysis. Related to Figure 4.

(A) TH counts from all eight batches pooled. Data is shown as boxplots ± SD. Wilcoxon T-test; ****p < 0.0001.

(B) Log2 fold change (FC) of TH expressed in early passage batches (B1-4) and late passage batches (B5-8).

(C) TH counts from early passage batches (B1-4). Pooled healthy control (WT) or GBA-PD lines are shown per batch. Data is shown as boxplots ± SD. Wilcoxon T-test; ns, not significant.

(D) TH counts from late passage batches (B5-8). Pooled healthy control (WT) or GBA-PD lines are shown per batch. Data is shown as boxplots ± SD. Wilcoxon T-test; ns, not significant.

Figure S4

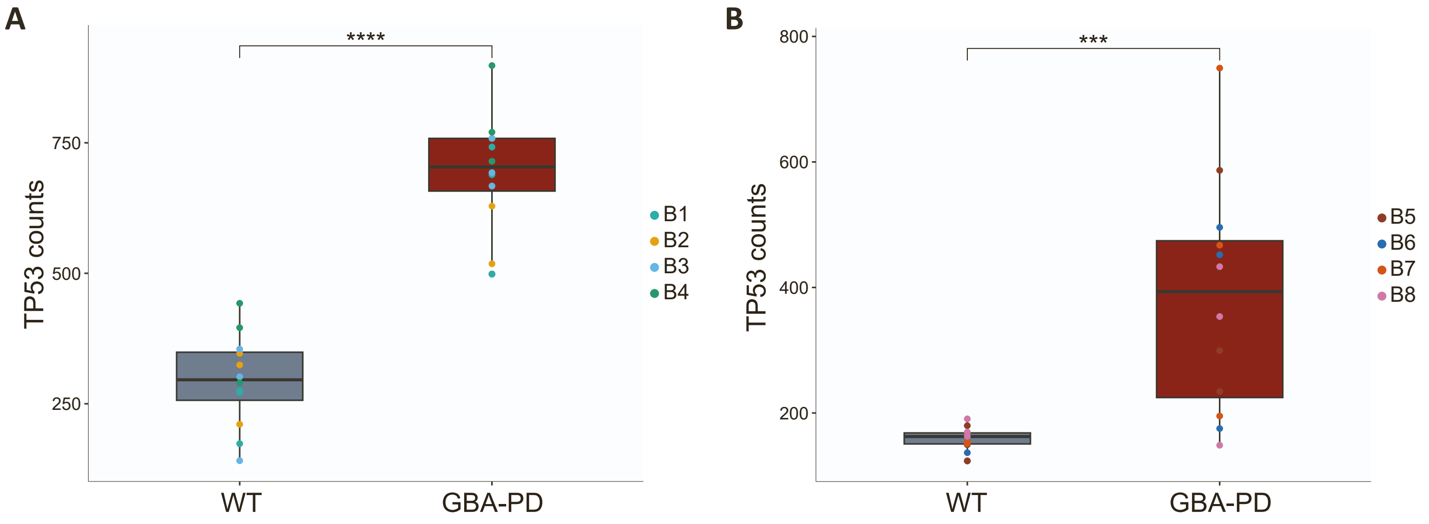

Figure S4: Transcriptomic analysis of senescence phenotype supports imaging analysis. Related to Figure 4.

(A) TP53 counts from early passage batches (B1-4) pooled. Data is shown as boxplots ± SD. Wilcoxon T-test; ****$p < 0.0001$.

(B) TP53 counts from late passage batches (B5-8) pooled. Data is shown as boxplots ± SD. Wilcoxon T-test; ***$p < 0.001$.

Figure S5

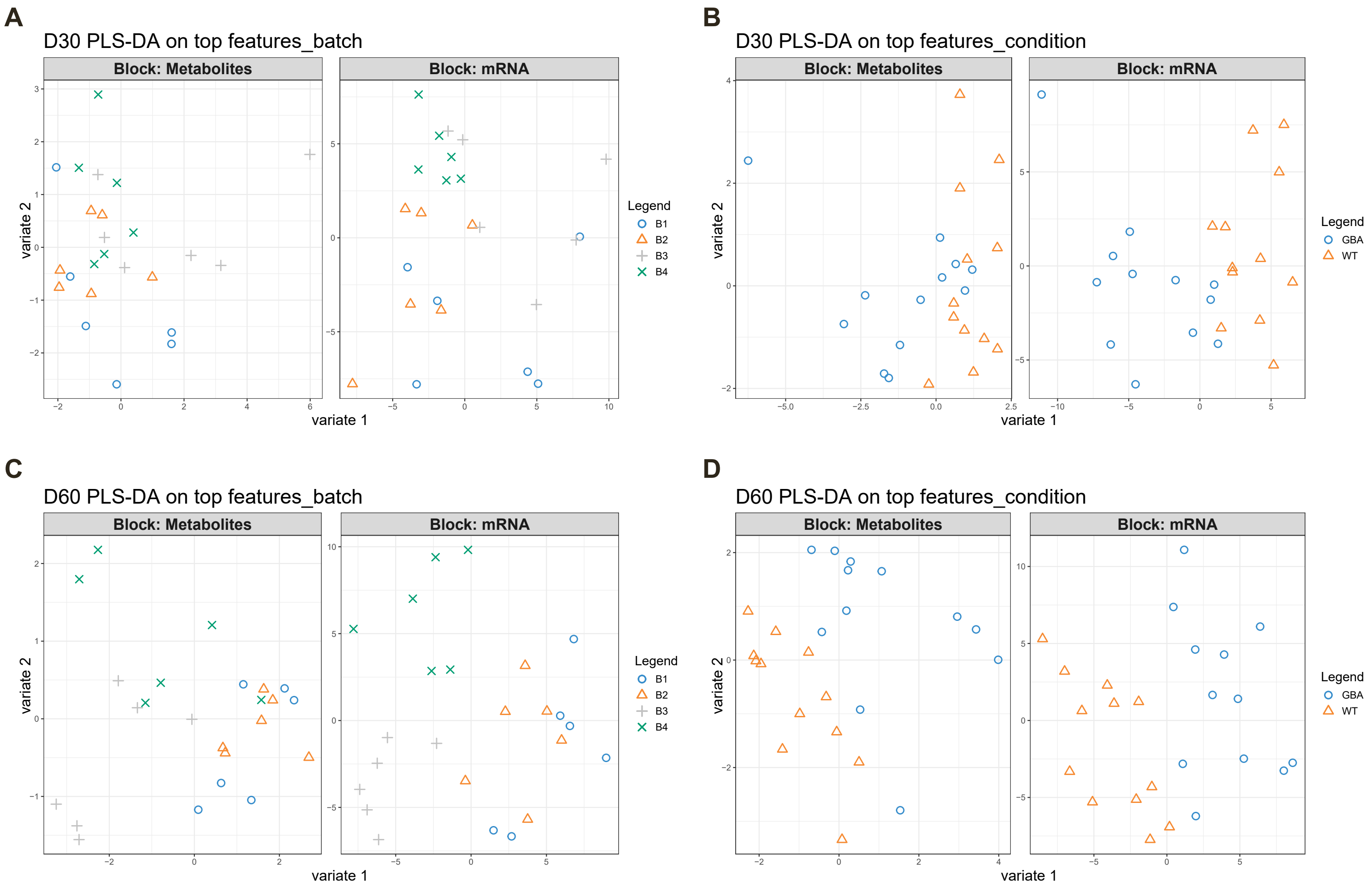
A
D30 PLS-DA on top features_batch
Block: Metabolites
Block: mRNA
variate 1
variate 2
Legend
B1
B2
B3
B4
B
D30 PLS-DA on top features_condition
Block: Metabolites
Block: mRNA
variate 1
variate 2
Legend
GBA
WT
C
D60 PLS-DA on top features_batch
Block: Metabolites
Block: mRNA
variate 1
variate 2
Legend
B1
B2
B3
B4
D
D60 PLS-DA on top features_condition
Block: Metabolites
Block: mRNA
variate 1
variate 2
Legend
GBA
WT

Figure S5: Partial Least Squares Discriminant Analysis (PLS-DA). Related to Figure 5.

(A) Day 30 sample discrimination by batch.

(B) Day 30 sample discrimination by condition.

(C) Day 60 sample discrimination by batch.

(D) Day 60 sample discrimination by condition.

Supplemental Tables

Table S1. Cell lines used in this study. Related to STAR Methods.

Sample ID	Diagnosis	Genotype	Sex	Age of onset	Age of sampling	Source of iPSC
WT1	Healthy	wt/wt	F	/	63	IBBL / Max Planck Institute
WT2	Healthy	wt/wt	F	/	68	IBBL / Max Planck Institute
WT3	Healthy	wt/wt	M	/	55	Coriell Institute
GBA1	PD	GBA-N370S/wt	F	77	81	European Bank for induced pluripotent Stem Cells
GBA2	PD	GBA-N370S/wt	F	55	55	University College London
GBA3	PD	GBA-N370S/wt	M	63	66	Coriell Institute

Table S2. Primary and secondary antibodies used for immunofluorescence stainings. Related to STAR Methods.

Antibody	Source	Cat. no.	RRID	Species	Dilution
TH	Abcam	ab76442	*AB_1524535*	Chicken	1:250
TH	Abcam	Ab112	*AB_297840*	Rabbit	1:1000
TUJ1	BioLegend	801201	*AB_2313773*	Mouse	1:300
53BP1	Novus BIologicals	NB100-304	*AB_10003037*	Rabbit	1:250
MAP2	Abcam	ab92434	*AB_2138147*	Chicken	1:250<br>1:1000
Anti-chicken 488	Jackson Immunoresearch	703-545-155	*AB_2340375*	Donkey	1:1000
Anti-chicken 647	Jackson Immunoresearch	703-605-155	*AB_2340379*	Donkey	1:1000
Anti-rabbit 488	Invitrogen	A21206	*AB_2535792*	Donkey	1:1000
Anti-rabbit 568	Invitrogen	A-10042	*AB_2534017*	Donkey	1:1000
Anti-mouse 647	Invitrogen	A-31571	*AB_162542*	Donkey	1:1000
